# Single‐cell RNA sequencing of retina revealed novel transcriptional landscape in high myopia and underlying cell‐type‐specific mechanisms

**DOI:** 10.1002/mco2.372

**Published:** 2023-09-20

**Authors:** Yunqian Yao, Zhenhua Chen, Qingfeng Wu, Yi Lu, Xingtao Zhou, Xiangjia Zhu

**Affiliations:** ^1^ Eye Institute and Department of Ophthalmology Eye & ENT Hospital Fudan University Shanghai China; ^2^ Key Laboratory of Myopia Chinese Academy of Medical Sciences National Health Center Key Laboratory of Myopia (Fudan University) Shanghai China; ^3^ Shanghai Research Center of Ophthalmology and Optometry Shanghai China; ^4^ State Key Laboratory of Molecular Development Biology Chinese Academy of Sciences Institute of Genetics and Developmental Biology Beijing China; ^5^ University of Chinese Academy of Sciences Beijing China; ^6^ Center for Excellence in Brain Science and Intelligence Technology Chinese Academy of Sciences Beijing China; ^7^ Chinese Institute for Brain Research Beijing China; ^8^ Beijing Children's Hospital Capital Medical University Beijing China; ^9^ Shanghai Key Laboratory of Visual Impairment and Restoration Shanghai China; ^10^ State Key Laboratory of Medical Neurobiology Fudan University Shanghai China

**Keywords:** glucose metabolism, high myopia, microglia, ON/OFF pathways, retina, single‐cell RNA sequencing

## Abstract

High myopia is a leading cause of blindness worldwide with increasing prevalence. Retina percepts visual information and triggers myopia development, but the underlying etiology is not fully understood because of cellular heterogeneity. In this study, single‐cell RNA sequencing analysis was performed on retinas of mouse highly myopic and control eyes to dissect the involvement of each cell type during high myopia progression. For highly myopic photoreceptors, *Hk2* inhibition underlying metabolic remodeling from aerobic glycolysis toward oxidative phosphorylation and excessive oxidative stress was identified. Importantly, a novel *Apoe* ^+^ rod subpopulation was specifically identified in highly myopic retina. In retinal neurons of highly myopic eyes, neurodegeneration was generally discovered, and the imbalanced ON/OFF signaling driven by cone‐bipolar cells and the downregulated dopamine receptors in amacrine cells were among the most predominant findings, indicating the aberrant light processing in highly myopic eyes. Besides, microglia exhibited elevated expression of cytokines and TGF‐β receptors, suggesting enhanced responses to inflammation and the growth‐promoting states involved in high myopia progression. Furthermore, cell–cell communication network revealed attenuated neuronal interactions and increased glial/vascular interactions in highly myopic retinas. In conclusion, this study outlines the transcriptional landscape of highly myopic retina, providing novel insights into high myopia development and prevention.

## Graphical abstract

Single‐cell RNA sequencing analysis revealed the metabolic remodeling from aerobic glycolysis toward oxidative phosphorylation and a specific degenerative rod subpopulation in highly myopic photoreceptors. Neurodegeneration was generally discovered in highly myopic retinal neurons with imbalanced ON/OFF signaling. Cell–cell communication network revealed attenuated neuronal interactions and increased glial/vascular interactions in highly myopic retinas, providing novel insights into its development and prevention.

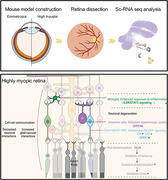

## 1 INTRODUCTION

In past decades, the prevalence of high myopia has rapidly elevated worldwide.^1^ High myopia has become a leading cause of blindness with dramatically increased risks of multiple ocular pathologies, including chorioretinal and retinal degeneration. Therefore, it is important to investigate the mechanisms underlying the development of high myopia.

Emerging evidence has suggested that the retina is involved in refractive development by sensing blurred images and triggering the signal transduction cascade of scleral remodeling and subsequent eye growth. Several genome‐wide association studies (GWAS) have also highlighted the essential roles of photosensitive and neuronal mechanisms underlying myopia progression.^2^, ^3^, ^4^ Previous studies involving pharmacological inhibition of retinal neurotransmitters or genetic knockdown of cell‐type‐specific genes (e.g., Gnat1^−/−^) confirmed that a variety of retinal cell types might play roles in refractive development.^5^, ^6^ However, the retina is a complex tissue in which multiple heterogeneous cell types form complicated communication networks. Studies performed on the whole retina may conceal critical contribution of individual cellular compartments to myopia.

The advent of single‐cell RNA sequencing (sc‐RNA seq) provides an opportunity to comprehensively investigate the complex biological systems. The single‐cell atlases of various ocular structures have been constructed.^7^, ^8^, ^9^, ^10^ We previously uncovered the single‐cell transcriptome atlas of lens epithelial cells, providing insight for pathogenesis and therapeutic targets for lens abnormalities.^9^ This technique enables detailed elucidation for retinal transcriptional landscape in high myopia and cell‐type‐specific roles in refractive development at single‐cell resolutions.

In this study, we applied sc‐RNA seq to the mouse retinas of highly myopic and contralateral control eyes. We reveal a high‐myopia‐specific degenerative rod subcluster and a decreased ratio of OFF‐cone‐bipolar cells (BCs) versus ON‐cone‐BCs, as well as pan‐neuronal degeneration in the highly myopic retinas. In addition, an enhanced response to inflammation is identified in the microglia of highly myopic retinas. These findings provide novel insights into the transcriptional landscape of highly myopic eyes and the retinal mechanisms underlying myopia development.

## 2 RESULTS

### 2.1 Identification of cell types affected by the development of high myopia

**FIGURE 1:**
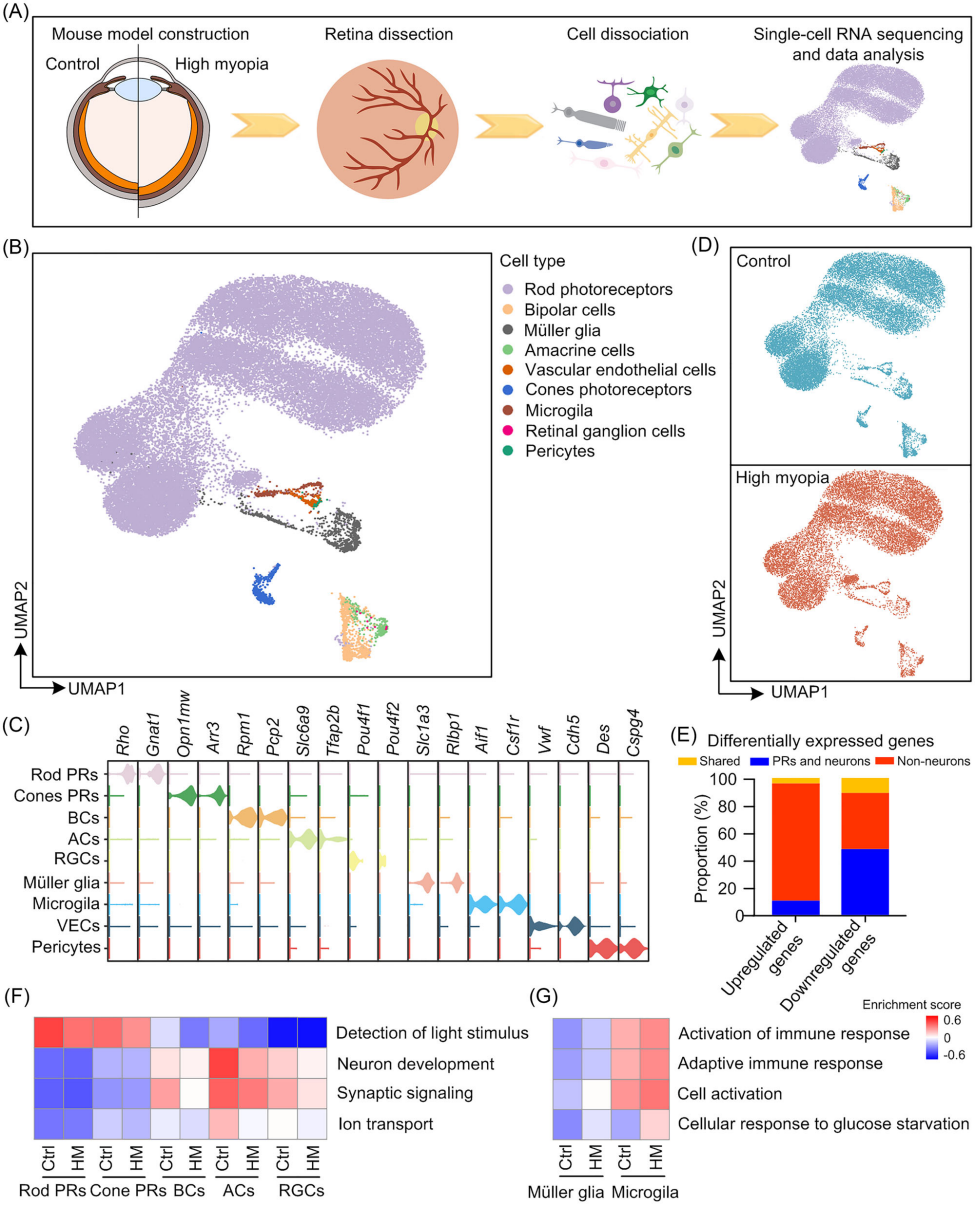
Single‐cell transcriptomic profiling of mice retinas in highly myopic and control eyes. (A) Experimental workflow for single‐cell RNA sequencing of mice highly myopic and control retina. (B) Uniform Manifold Approximation and Projection (UMAP) visualization of retinal transcriptional landscape, colored according to cell types. (C) Specific expression of cell type marker genes in each cell type. (D) UMAP visualization of retinal transcriptional landscape, colored according to groups (high myopia and control). (E) The distribution of differentially expressed genes (DEGs) (summarized from DEGs calculated within each cell type between highly myopic and control eyes); “Photoreceptors (PRs) and neurons” include rod photoreceptors, cone photoreceptors, bipolar cells, amacrine cells, and retinal ganglion cells; “non‐neurons” include Müller glial cells, microglial cells, vascular endothelial cells, and pericytes; “shared” means the genes upregulated or downregulated in both “PRs and neurons” and “non‐neurons.” (F) Gene set variation analysis (GSVA) for pathways of interest in PRs and neurons. (G) GSVA for pathways of interest in Müller glial cells and microglial cells. BC, bipolar cell; AC, amacrine cell; RGC, retinal ganglion cell; VEC, vascular endothelial cell; HM, high myopia; Ctrl, control.

To explore the involvement of retinal cell types in the development of high myopia, sc‐RNA seq was performed on retinas dissected from highly myopic mice models (Figure 1A). The models were established by placing a −25.00 diopter (D) lens on the right eyes for 4 weeks and the left eyes served as self‐controls. There was no significant difference in the refractive status between the two groups at the start of the experiments. After modeling, the right eyes exhibited a significant myopic shift compared with the control eyes (−1.07 D vs. 7.72 D, *p* < 0.001, paired *t*‐test).

A total of 15,335 cells from highly myopic retinas and 15,991 cells from self‐control retinas were analyzed, respectively (Figure S1A). The cell transcriptomes from all the samples were integrated and analyzed together to gain power for detecting rare cell types. Based on the expression profiles of the top 2000 highly variable genes, we initially identified 11 transcriptionally distinctive clusters and determined main retinal cell types (rod photoreceptors [Rho, Gnat1], cone photoreceptors [Opn1mw, Arr3], BCs [Trpm1, Pcp2], amacrine cells [ACs] [Slc6a9, Tfap2b], retinal ganglion cells [RGCs] [Pou4f1, Pou4f2], Müller glial cells [Slc1a3, Rlbp1], microglia [Aif1, Csf1r], vascular endothelial cells [Vwf, Cdh5], and pericytes [Des, Cspg4]) using well‐established cell type markers based on secondary clustering (Figure 1B,C and Figure S1B).^11^ The general distribution of cell types did not differ significantly between the highly myopic and control eyes (Figure 1D), suggesting that abnormal gene expression in each cell type might be more important to the pathogenesis of high myopia compared with changes in the proportions of cell types. Therefore, we next explored the cell‐type‐specific alterations in highly myopic retinas.

We compared the transcriptional signatures of each retinal cell type between highly myopic and control eyes. We found that the highest percentage of upregulated genes was enriched in non‐neuronal cells, whereas the downregulated genes were mostly enriched in neuronal cells (Figure 1E). The downregulated genes in neuronal cells were involved in neuron development, synaptic signaling, regulation of ion transport, and detection of light stimulus (Figure 1F), consistent with previous large‐population GWAS analyses which revealed that visual perceptual and neuronal mechanisms contributed to the development of myopia.^2^, ^3^, ^4^ By comparison, the upregulated genes in glia were enriched for cell activation and immune‐related processes (Figure 1G).

We also scored each retinal cell type according to the expression of myopia candidate genes (Figure S2),^2^ and we observed significantly higher scores in BCs and ACs and comparable scores in other cell types (Figure S1C). Overall, these results indicate that multiple mechanisms, especially neuronal mechanisms, might be involved in the development of high myopia. The cell‐type‐specific changes and mechanisms need to be further investigated.

### 2.2 Identification of a specific degenerative rod subcluster in highly myopic eyes

**FIGURE 2:**
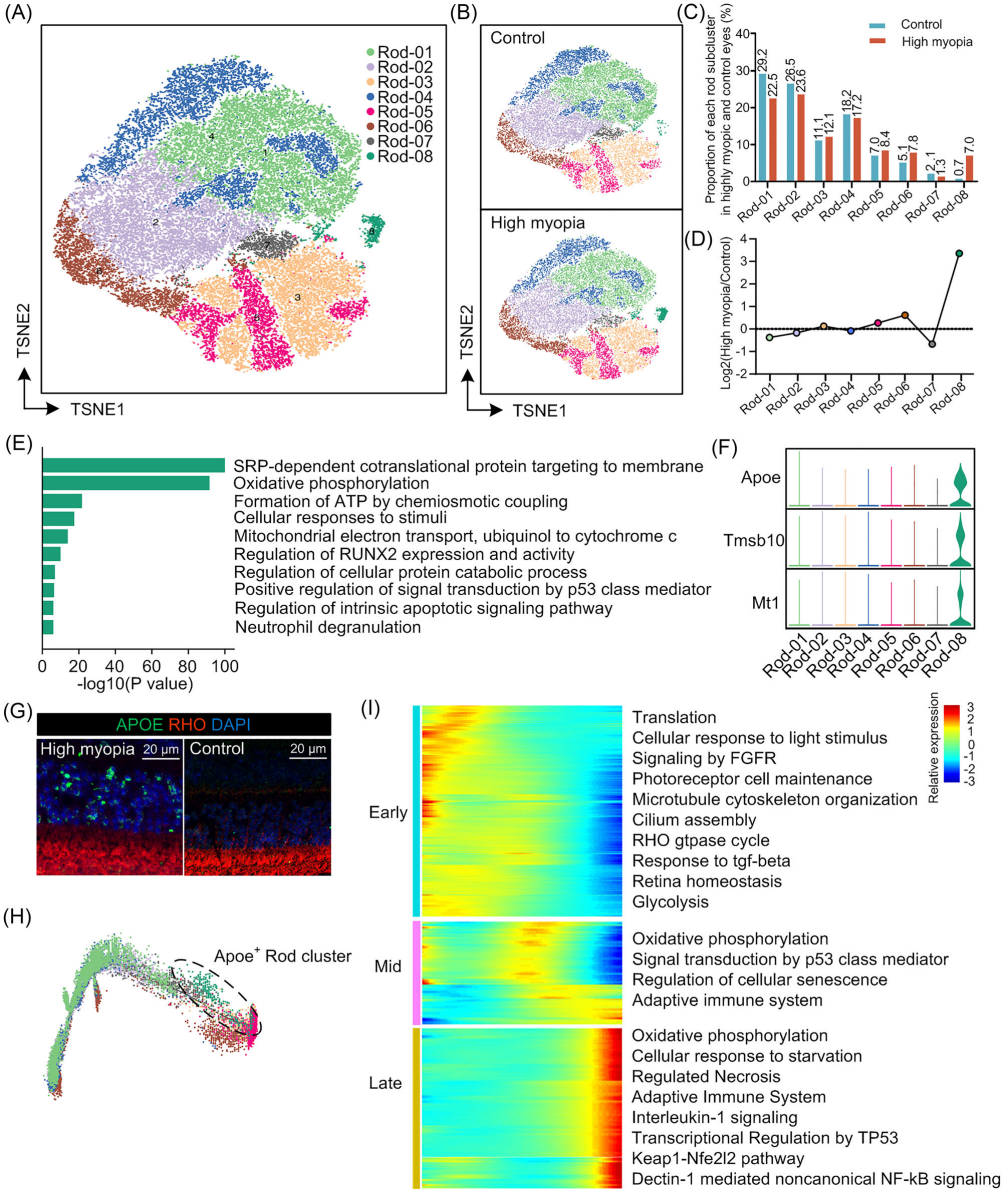
Specific subcluster of rod photoreceptors identified in highly myopic eyes. (A) t‐Distributed Stochastic Neighbor Embedding (TSNE) visualization of rod photoreceptors. (B) TSNE visualization of rod photoreceptors in highly myopic and control eyes, respectively. (C) Relative proportion of each rod subcluster in highly myopic and control eyes. (D) The fold change of relative proportions of each rod subcluster between highly myopic and control eyes. (E) Pathway enrichment analysis results of the marker genes for rod subcluster 8. (F) Marker genes of rod subcluster 8. Apoe was the top marker gene, therefore, the rod subcluster 8 was named as “Apoe^+^ rod photoreceptors”. (G) Immunofluorescence staining to confirm the specific existence of Apoe^+^ rod photoreceptors in highly myopic retina. (H) The degenerative trajectory of rod photoreceptors where the subcluster 8 appeared at the late period. (I) Heat map showing the dynamic expression profiles of genes along the trajectory and the representative pathways enriched for each pseudotime period.

Several studies have indicated that rod and cone photoreceptors are involved in regulating ocular growth.^12^, ^13^, ^14^ Recent investigations in monkeys showed that photoreceptors play important roles in modulating eye growth by producing signals toward retinal pigment epithelial (RPE) cells and promote scleral tissue remodeling.^12^, ^13^, ^15^, ^16^ However, the understanding of cellular heterogeneity on rod photoreceptors remained limited. To explore the heterogeneous changes in high myopia, we divided the population of rod photoreceptors into eight subpopulations (Figure 2A). Of note, subcluster 8 was specifical to highly myopic eyes (Figure 2B–D). Based on the transcriptional signature, the functional enrichment analysis showed that this high‐myopia‐specific rod subcluster was related to oxidative phosphorylation, ATP formation, response to stimuli, and intrinsic apoptotic signaling pathway (Figure 2E), indicating a specific mode of rod degeneration in highly myopic retinas. Because *Apoe* was the top marker gene that was highly expressed in subcluster 8 (Figure 2F), we named subcluster 8 the *Apoe* ^+^ rod subcluster. Immunofluorescence staining further confirmed the specific existence of *Apoe* ^+^ rod photoreceptors in highly myopic retinas (Figure 2G). The morphology of *Apoe* ^+^ cells in the outer nuclear layer of the retina was similar to that of common rod photoreceptors and the *Apoe* ^+^ cell count was significantly higher in highly myopic retinas than in control eyes (Figure 2G).

Next, we endeavored to uncover the biological significance of *Apoe* ^+^ photoreceptors in promoting myopia development. Using the Monocle2 package, we constructed an unsupervised pseudotime trajectory based on gene signatures of all rod photoreceptors without prior clustering information. In this analysis, the *Apoe* ^+^ rod subcluster was located at one end of the trajectory (Figure 2H). Because this subcluster was associated with stress and degeneration, we considered this trajectory to represent the neurodegenerative process, and the opposite end was defined as the starting point. Notably, genes related to normal photoreceptor functions and metabolic activities (photoreceptor cell maintenance, cilium assembly, and glycolysis) were significantly downregulated along the trajectory, whereas genes enriched for pathological states and altered metabolic characteristics (cellular response to hypoxia/starvation, inflammatory responses, and oxidative phosphorylation [OXPHOS]) were upregulated during the late period (Figure 2I).

**FIGURE 3:**
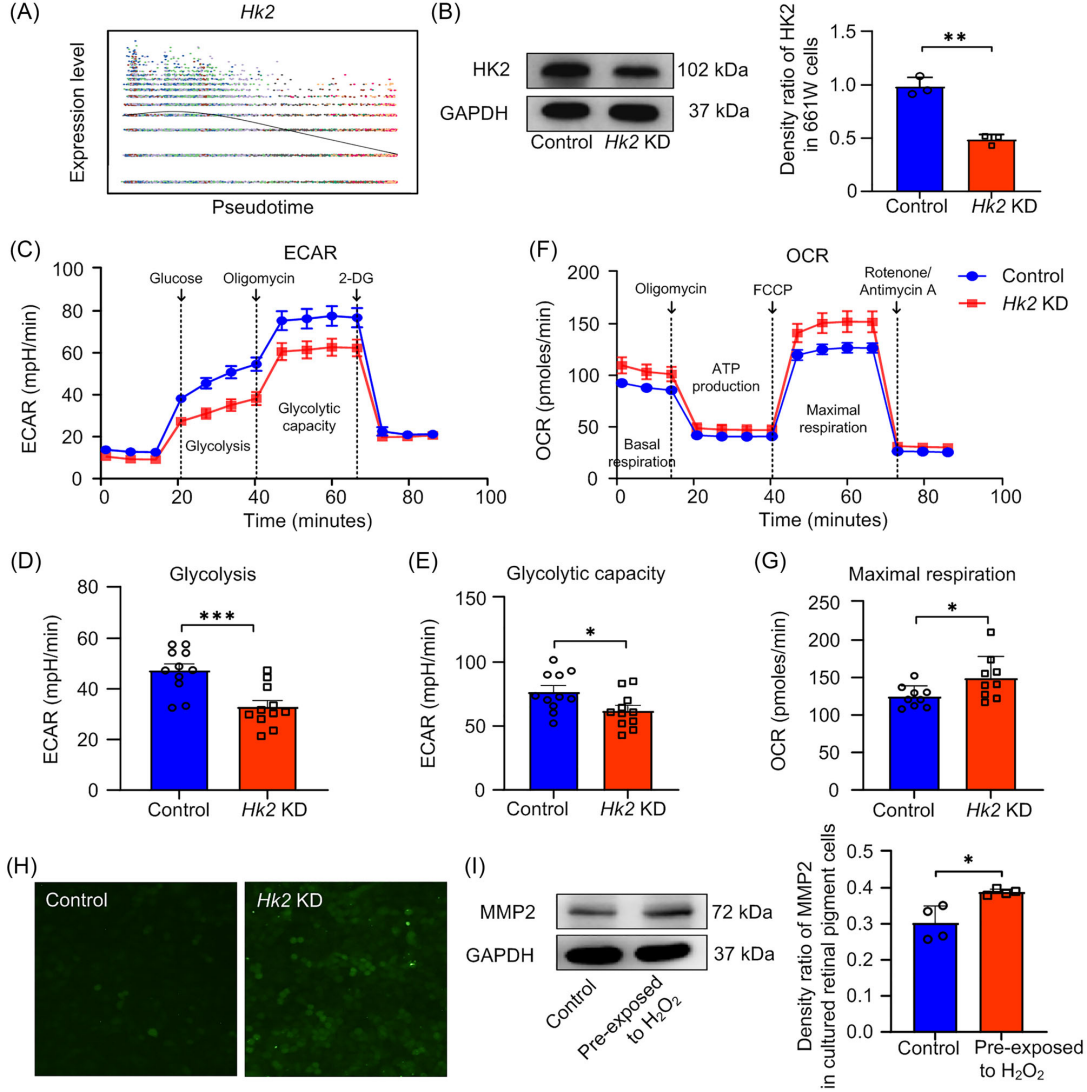
Effect of *Hk2* knockdown (KD) on photoreceptor cells. (A) The decreased expression of *Hk2* along the pseudotime trajectory. (B) Successful knockdown of *Hk2* in a photoreceptor cell line confirmed by western blotting. (C–E) Seahorse metabolic analysis (glycolysis stress tests) of control cells and Hk2 KD cells. (F–G) Seahorse metabolic analysis (mitochondria stress tests) of control cells and *Hk2* KD cells. The results are presented as the mean ± SEM. (H) Increased reactive oxygen species (ROS) production in the photoreceptor cells after *Hk2* knockdown stained with a ROS detection kit. (I) Increased expression of MMP2 in mouse primary retinal pigment epithelial (RPE) cells co‐cultured with photoreceptor cells, which were pre‐exposed to H_2_O_2_. The control group was co‐cultured with photoreceptor cells without any treatment. ECAR, extracellular acidification rates; OCR, the oxygen consumption rates.

Notably, the expression level of *Hk2*, the first rate‐limiting enzyme of glycolysis in photoreceptors, was significantly downregulated along the trajectory (*q* value = 5.02 × 10^−80^) (Figure 3A). To evaluate the metabolic outcomes of *Hk2* deficiency, glycolysis and OXPHOS levels were measured using the Seahorse metabolic analyzer. After *Hk2* knockdown (Figure 3B), the extracellular acidification rates (ECAR) exhibited a reduction in glycolysis and glycolytic capability (Figure 3C–E), whereas the oxygen consumption rates (OCR) exhibited an increase in maximal respiration compared with control cells (Figure 3F–G), indicating *Hk2* deficiency caused a metabolic shift from glycolysis to OXPHOS, and that *Hk2* is required for maintaining cellular glycolytic activities in photoreceptors. *Hk2* knockdown also led to increased formation of reactive oxygen species (ROS) (Figure 3H). ROS is closely linked with myopia.^17^ To evaluate the role of ROS in photoreceptors on myopia development, RPE cells were co‐cultured with photoreceptor cells pre‐exposed to H_2_O_2_ and exhibited a significant upregulation of the myopia molecular marker, MMP2 (Figure 3I). Taken together, our results suggest that oxidative stress induced by glucose metabolism remodeling in photoreceptors in the context of blurred vision might trigger phototransduction dysfunction and progression of myopia.

### 2.3 Decreased neuronal excitability of interneurons and imbalanced BC‐driven ON/OFF light processing signaling in highly myopic eyes

**FIGURE 4:**
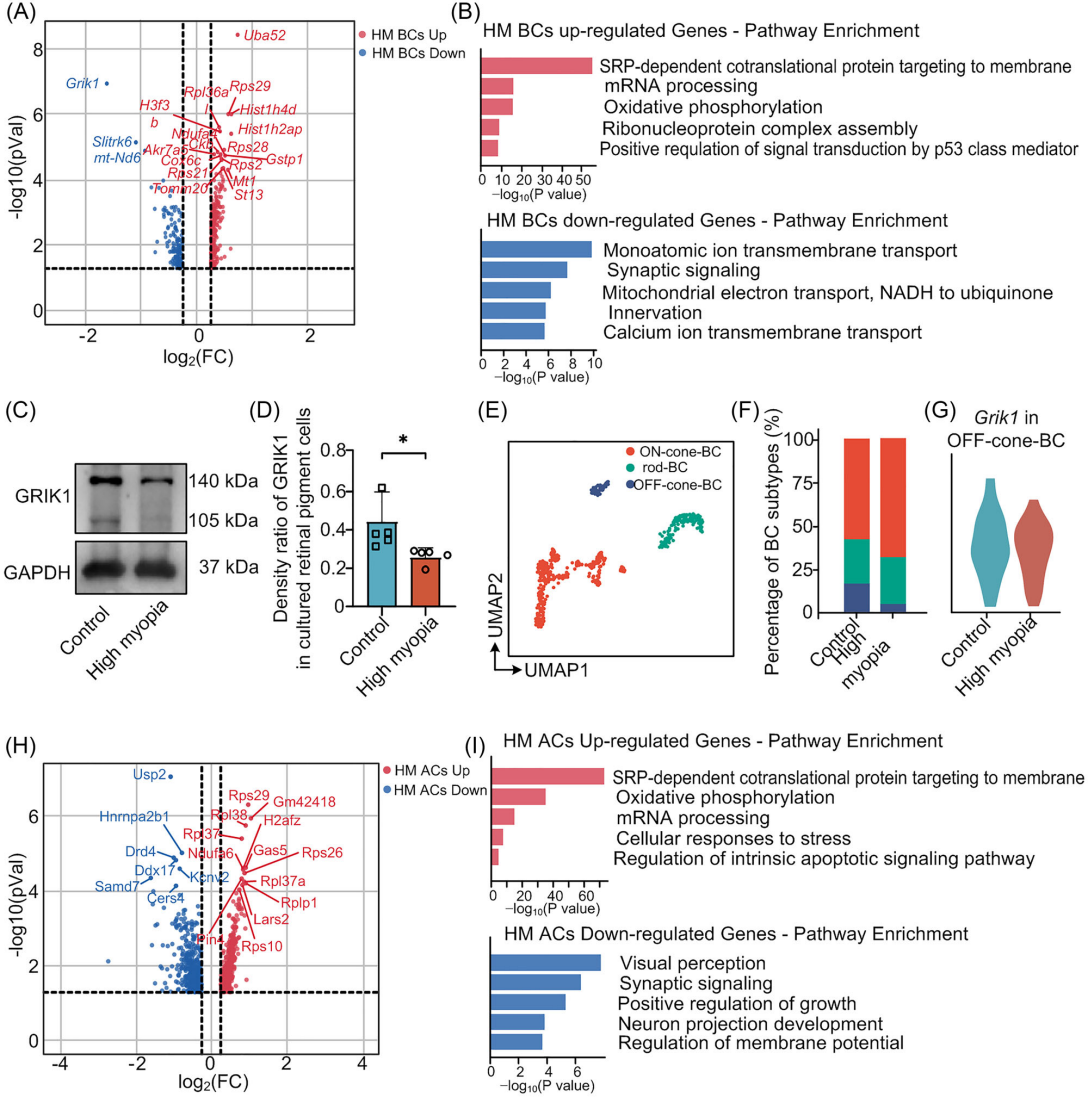
Decreased neuronal excitability of interneurons and imbalanced bipolar cell (BC)‐driven ON/OFF light processing signaling in highly myopic eyes. (A) Volcano plot displaying differentially expressed genes detected between highly myopic BCs and control BCs. (B) Pathway enrichment analysis results of upregulated and downregulated genes in highly myopic BCs. (C and D) Downregulated protein level of GRIK1 in highly myopic retinas. (E) UMAP visualization of all BCs, colored according to cell subtypes. (F) Proportion of each BC subtype in highly myopic and control eyes. (G) Downregulated expression of *Grik1* in highly myopic OFF‐cone‐BCs. (H) Volcano plot displaying differentially expressed genes detected between highly myopic amacrine cells (ACs) and control ACs. (I) Pathway enrichment analysis results of upregulated and downregulated genes in highly myopic ACs. HM, high myopia/highly myopic.

BCs anatomically connect the outer and inner layers of the retina and transmit signals from photoreceptors to RGCs. Previous studies have suggested that altered BC signaling transduction is involved in myopia development^18^, ^19^; however, the underlying cell‐type‐specific molecular mechanisms remain largely unknown. To identify the role of BC signaling transduction, we first surveyed the differentially expressed genes between highly myopic and control BCs (Figure 4A). Surprisingly, we observed remarkable neurodegenerative manifestations characterized by multiple downregulated pathways related to synapses and neuron development (Figure 4B). Notably, *Grik1*, which expresses an ionotropic glutamate receptor, was the top downregulated gene in highly myopic BCs (Figure 4A). Because *Grik1* was enriched in BCs (Figure S2), western blotting was performed at the tissue level and confirmed that GRIK1 was downregulated in highly myopic BCs (Figure 4C,D). These results were consistent with those of previous GWAS meta‐analyses, which identified GRIK1 as a myopia candidate gene in a large cohort of more than 250,000 participants.^2^ Therefore, these results highlight the role of aberrant BC‐mediated light processing in the development of myopia by regulating the expression of glutamate receptors.

To further investigate the transcriptional alterations of highly myopic BCs, we performed unsupervised subclustering of these cells and classified them into three subpopulations, rod‐BCs (*Prkca* ^+^), ON‐cone‐BCs (*Scgn* ^+^ *Isl1* ^+^), and OFF‐cone‐BCs (*Scgn* ^+^ *Isl1* ^−^), based on the expression of established subtype markers (Figure 4E).^20^ We first surveyed the proportional variance of each cell type and observed an increased proportion of ON‐cone‐BCs and a decreased proportion of OFF‐cone‐BCs in highly myopic retinas than in control eyes, whereas the proportion of rod‐BCs was comparable between highly myopic and control eyes (Figure 4F and Figure S3A). This result further implied the role of imbalanced ON–OFF signaling (especially cone‐BC‐driven ON–OFF signaling) in promoting high myopia development. In addition, we investigated the expression profile of *Grik1* in BC subpopulations and found that it was specifically expressed in OFF‐cone‐BCs (Figure S3B), and its expression was also decreased in highly myopic OFF‐cone‐BCs (Figure 4G). These results provide additional support for the role of glutamate receptor regulation in the development of high myopia.

The differential analyses were extended to ACs that exhibited similar trends toward increased responses to stress and decreased expression of genes related to neuronal function and synaptic signaling (Figure 4H,I). Notably, *Drd4* was among the top downregulated genes in highly myopic ACs (Figure 4H). As a component of D2‐like dopamine receptors (D2Rs), *Drd4* could actively respond to the environmental lighting by modulating intracellular Ca^2+^ levels and therefore regulating Ca^2+^‐dependent signaling processes.^21^ This result supports previous observations suggesting that D2Rs, rather than D1‐like receptors (D1Rs), mediate the effect of dopamine for protecting against lens‐induced myopia.^22^, ^23^ In summary, these findings strongly suggest that abnormal BC/AC‐mediated light processing within the retina may be the vital factor that triggers and maintains myopia development.

### 2.4 Highly myopic eyes showed enhanced responses to inflammatory and growth factors

**FIGURE 5:**
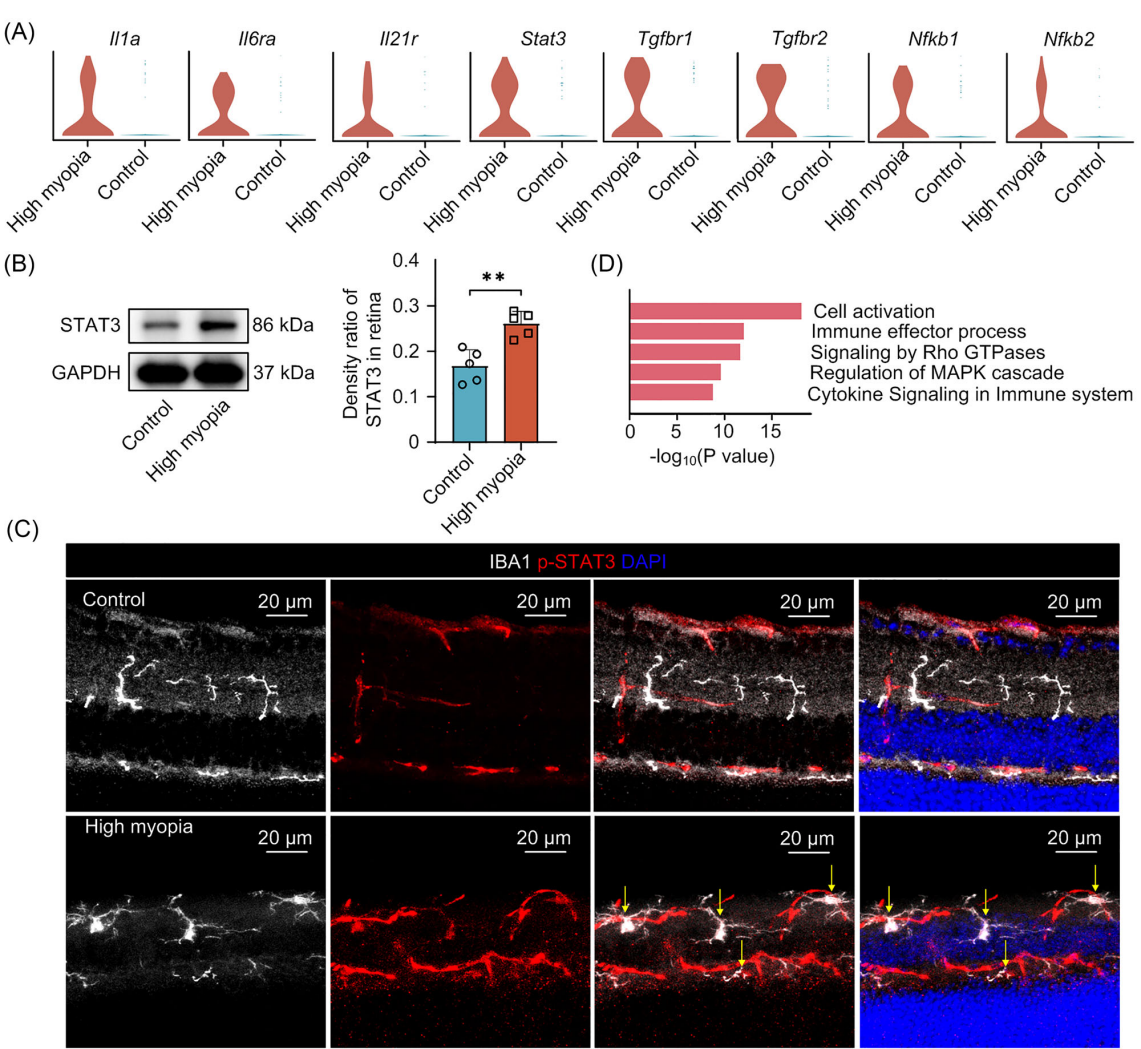
Enhanced responses to inflammatory and growth factors in highly myopic eyes. (A) Violin plot displaying upregulated cytokine and growth factor receptor genes in highly myopic microglia. (B) Up‐regulation of STAT3 in highly myopic retina confirmed by western blotting. (C) Immunofluorescence staining of IBA1 and phosphorylated‐STAT3 (p‐STAT3). (D) Pathway enrichment analysis for upregulated genes in highly myopic microglial cells.

Accumulated evidence supports the potential involvement of inflammation in high myopia progression.^24^, ^25^, ^26^ For instance, previous studies have reported elevated levels of interleukin (IL)‐6 and transforming growth factor (TGF)‐β in the ocular environment of highly myopic patients.^24^, ^25^ To dissect the inflammatory profiles of highly myopic eyes at a single‐cell resolution, we determined the expression levels of cytokines, growth factors, and the corresponding receptors in microglia, the retina‐resident immune cells. We found that *Il1a*, *Il6ra*, *Il21r*, *Tgfbr1*, and *Tgfbr2* and downstream transcriptional regulators (*Stat3*, *Nfkbr1*, and *Nfkbr2*) were significantly increased in the microglia of highly myopic eyes (Figure 5A). These results suggested these eyes exhibited enhanced responses to cytokines and growth factors, as well as a possible activation of the STAT3 signaling pathway. Western blotting confirmed the increased STAT3 expression in highly myopic retinas (Figure 5B). Double‐label immunofluorescence staining of phosphorylated‐STAT3 (p‐STAT3) and IBA1 further showed the activation of STAT3 signaling in highly myopic retinal microglial cells (Figure 5C). Meanwhile, genes enriched for cell activation, cell migration, and cellular responses to stress were also upregulated in highly myopic microglia (Figure 5D). Collectively, these findings provide novel evidence for the potential involvement of enhanced microglial responses to inflammation and the growth‐promoting states in high myopia progression.

### 2.5 High myopia alters retinal cell‒cell communication

**FIGURE 6:**
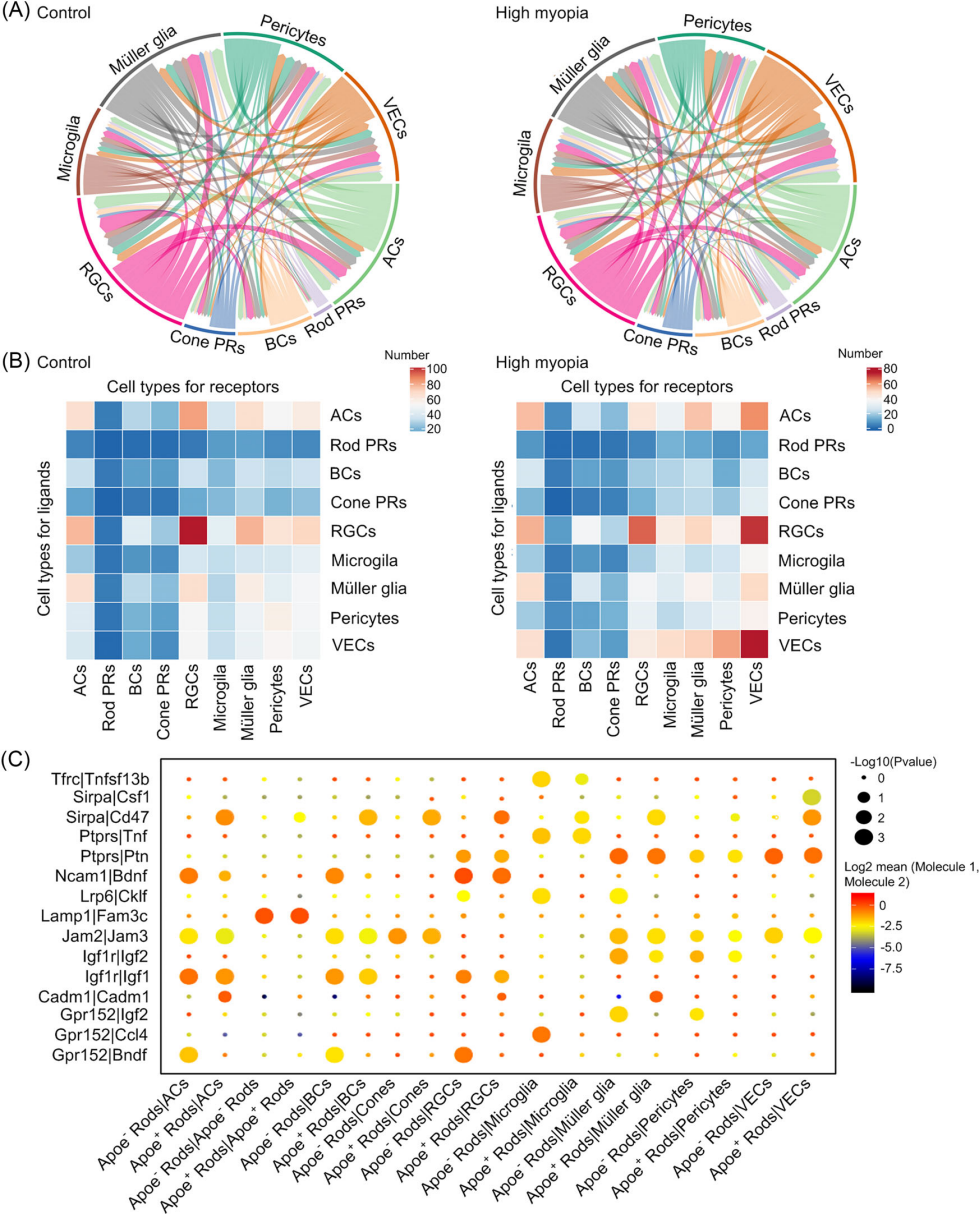
High myopia alters retinal cell–cell communication. (A) Chord diagrams of cellular interactions among all cell types in highly myopic and control retinas, separately. (B) Heatmaps exhibiting the interaction profiles between every two cell types in highly myopic and control retinas, separately. (C) Dot plot of ligand‐receptor interactions between Apoe− or Apoe+ rod photoreceptors and other cell types. BCs, bipolar cells; ACs, amacrine cells; RGCs, retinal ganglion cells; VECs, vascular endothelial cells.

The retina is composed of multiple cell types, which form a complex regulatory network for light processing through synaptic transmission and interactions via ligand‒receptor pairs or adhesive molecules. To investigate the alterations of cell‒cell interactions in highly myopic retinas, the cell‒cell communication network was constructed in highly myopic and control retinas based on the expression profiles of ligand‒receptor pairs in each cell type (Figure 6A). A total of 2651 interactions among cell types were observed in the control retinas, compared with 2136 were found in highly myopic retinas. Of note, the interactions within photoreceptors or neurons were attenuated or eliminated, whereas those between glial or vascular cells and other cell types were remarkably increased in highly myopic retinas (Figure 6B), consistent with our prior observations of degenerative neurons and activated glia in highly myopic eyes.

Furthermore, because we identified a specific subcluster of rod photoreceptors (*Apoe* ^+^ rods) in highly myopic eyes, we explored the altered interactions associated with *Apoe* ^+^ rods (Figure 6C). Notably, these cells showed downregulated expression of brain‐derived neurotrophic factor (*Bdnf*) receptors (*Ncam1* or *Gpr152*) and insulin‐like growth factor 1 (*Igf1*) receptor (*Igf1r*); these changes may hinder the cells’ ability to receive nutritional support from the surrounding neurons (Figure 6C). Besides, the overexpression of *Sirpa* in *Apoe* ^+^ rods may enhance the interactions between *Apoe* ^+^ rods and all other retinal cell types which expressed *Cd47* (Figure 6C). While recent studies have reported the important roles of *Cd47*‐*Sirpa* ligand‐receptor pair in regulating synapse strengthening and neuron survival,^27^ this result implicated the retinal neuronal network undergoing remodeling in highly myopic eyes. Taken together, this analysis of the cell–cell communication network revealed attenuated neuronal interactions and increased glial/vascular interactions in highly myopic retinas, and these aberrant communication patterns provide novel insight for understanding the pathogenesis of myopia.

## 3 DISCUSSION

High myopia is becoming increasingly prevalent, and it has been estimated that it will affect 10% of the world's population by 2050.^28^ There is sufficient evidence to suggest that the retina triggers the development of myopia by perceiving aberrant visual experience and initiating the signaling cascade that finally transmits to the myopia target tissue, the sclera. However, owing to the heterogeneity of retinal cell types, the contribution of individual retinal pathways to myopia development remains largely unknown. In this study, we explored the single‐cell transcriptional signatures of highly myopic and control retinas and identified cell‐type‐specific mechanisms underlying the pathogenesis of high myopia that included high‐myopia‐specific degenerative rod photoreceptor subpopulation, imbalanced ON‐BC/OFF‐BC signaling, and decreased retinal neuronal activity. Moreover, microglia activity was increased in high myopic retinas, suggesting that metabolic and inflammatory factors play roles in the development of high myopia.

The retina processes light information through a neuronal signaling cascade and maintains its homeostasis via glia and vascular cells. Photoreceptors have been implicated in the pathogenesis of abnormal refractive development. Tin et al. reported that “abnormal photoreceptor inner segment morphology” was the most significant gene set enriched for myopia‐associated genes; other photoreceptor‐related gene sets include the “thin retinal outer nuclear layer”, “detection of light stimulus”, and “nonmotile primary cilium”.^2^ Previous functional studies have also confirmed the attenuation of photoreceptor cell responses to flash electroretinography (a‐wave and d‐wave) in a murine model of myopia.^6^ However, the underlying molecular mechanism was largely unknown.

Photoreceptors consume a large amount of energy for phototransduction and constant renewal of their outer segments, but they preferentially consume glucose through aerobic glycolysis despite possessing abundant mitochondria and enzymes for OXPHOS,^29^, ^30^, ^31^ sharing similar metabolic characteristics to cancer cells, in a process known as the Warburg effect. Because photoreceptors are the most abundant cells in the retina, their metabolic changes would have a remarkable impact on the retinal microenvironment. In this study, we revealed a shift toward OXPHOS in highly myopic photoreceptors. Similar metabolic alternations in myopic eyes have also been also mentioned in previous studies, which used bulk RNA sequencing of retinas.^6^, ^32^

More importantly, we identified the involvement of *Hk2* deficiency in this metabolic remodeling process. Hexokinases (HKs) are the first rate‐limiting enzymes in glycolysis, and HK2 is the main subtype expressed in photoreceptors.^33^ In this study, *Hk2* was among the top downregulated genes along the rod degenerative trajectory in highly myopic eyes. Silencing *Hk2* led to a decreased glycolysis and an increased in OXPHOS in a photoreceptor cell line using Seahorse metabolic analysis. This result was consistent with those performed in a hepatocellular carcinoma cell line by DeWaal et al. and those in *Hk2* mutant mice by Zhang et al.^33^, ^34^ Importantly, *Hk2* knockdown contributed to ROS as possible by‐products of excessive OXPHOS in mitochondria, because HK2 could help to limit ROS generation by maintaining local ADP levels.^35^, ^36^ ROS is closely linked with myopia.^17^ It may induce upregulation of HIF‐1α or proinflammatory cytokines, which have been elaborated to promote myopia development.^26^, ^37^, ^38^ This may explain why ROS induced in photoreceptors further upregulated the expression of myopic molecular marker (MMP2) in co‐cultured RPE cells in our study, and therefore indicate how HK2‐related glucose metabolism might be related to high myopia development. Taken together, our findings demonstrate that the oxidative environment derived from an aberrant metabolic shift toward OXPHOS in the retina may help trigger the signaling transmission cascade toward the sclera, providing a new perspective for understanding the development of high myopia and the early‐onset of retinal degeneration in highly myopic eyes.

Another important finding is the discovery of a novel rod photoreceptor subcluster characterized by APOE expression in highly myopic retinas. APOE is critical for transporting cholesterol and lipids in the central nervous system, and it is strongly associated with neuronal degenerative diseases and aging.^39^ Previous studies have demonstrated neuronal APOE production after acute stress and injury, including oxidative stress, traumatic brain injury, and amyloid‐β (Aβ) accumulation.^40^, ^41^, ^42^ Therefore, these specific *Apoe* ^+^ rod photoreceptors might be related to the neuropathologic microenvironments of highly myopic retinas, playing roles in regulating cholesterol redistribution for cellular repair. The specific factors underlying these observations remain unclear; therefore, further functional mechanistic studies are necessary.

An interesting finding of this study was the imbalanced ON‒OFF signaling systems mediated by BCs in highly myopic eyes. Accumulative evidence has suggested the involvement of BC‐mediated light processing in the development of myopia.^18^, ^19^, ^43^ Hendriks et al. investigated the incidence and degree of myopia in inherited retinal dystrophies with genetical degeneration of a specific cell type and found that BC dysfunction was associated with the highest risk of high myopia.^18^ In our study, the proportion of OFF‐cone‐BCs was decreased. We also observed downregulation of *Grik1* in highly myopic OFF‐cone‐BCs. In support, Jiang et al. found that OFF‐cone‐BC signaling was highly associated with a myopia‐susceptibility locus which genotyped between 186 twin volunteers,^19^ providing additional evidence for OFF‐cone‐BC pathways in myopia development. The ON and OFF visual systems are established at the first retinal synapses (the photoreceptor‒BC synapses) and separated throughout the downstream retinal information processing systems (BC‒AC and BC‒RGC synapses). They respond to light stimuli in opposite ways because ON‐BCs depolarize, whereas OFF‐BCs hyperpolarize in response to light. It is predicted that aberrant visual experience would trigger glutamate‐receptors‐mediated ON/OFF‐BC signaling imbalance, manifested as altered contrast sensitivity and spatial resolution, directly leading to changes in retinal dopamine metabolism and choroidal thickness as eyes are attempting to reduce image defocus on the retina,^43^, ^44^ and therefore promoting or maintaining high myopia progression. Further investigations using animal models with conditional knockout of interested genes (e.g. *Grik1*) in BCs may provide more evidence.

Neuronal dysfunction in the light processing pathway also extended to ACs. Emerging evidence has revealed a role of ACs in ocular refractive development. Pan et al. reported that deregulated phosphorylation modification of connexin36 in aII ACs contributed to the development of myopia.^28^ In our study, we identified significant dysfunction of ACs, including downregulated synaptic signaling transmission and neuron development, implicating impaired AC function in complex retinal image processing, especially in the adjustment of image brightness, which is strongly associated with the onset of myopia. Furthermore, because ACs are the main retinal neurons that possess a dopaminergic synaptic system, our finding that the D2‐like dopamine receptor *Drd4* was significantly downregulated in highly myopic ACs provides further support for the widely acknowledged myopia development theory involving dopamine signaling.^45^

Microglia are the main resident immune cells in the retina. Neuroinflammation mediated by microglia plays vital roles in retinal pathologies. Accumulative evidence has suggested that inflammation is involved in the development of myopia. Lin et al. reported a higher incidence of myopia among patients with inflammatory diseases, and elevated expression levels of c‐Fos, nuclear factor‐κB, and IL‐6 in murine myopic eyes.^26^ However, in our study, we found that a group of cytokine receptors, rather than cytokines, were significantly elevated. While proinflammatory cytokines could be derived from other cell types, such as blood‐derived macrophages that infiltrated the retina, our results highlight the enhanced responses of highly myopic eyes to proinflammatory environment. Notably, the STAT3 signaling pathway was activated in highly myopic microglia characterized by elevated p‐STAT3 expression, exhibiting an aging or neuroinflammatory profile.^46^, ^47^ Because this activation might be a response to the pathological environment in highly myopic retinas or an amplifier to exacerbate myopia progression via regulating myopia‐related genes, further studies are needed to dissect the causal relationship between these two events.

Given that RPE and choroid also play important roles in scleral remodeling and eye growth, one limitation of this study is the absence of RPE transcriptomic data as is rarely captured in retinal sc‐RNA‐seq samples.^48^ Further RNA sequencing of RPE/choroid is expected to elucidate the role of retina‐RPE/choroid‐sclera system on the regulation of myopia development.

**FIGURE 7:**
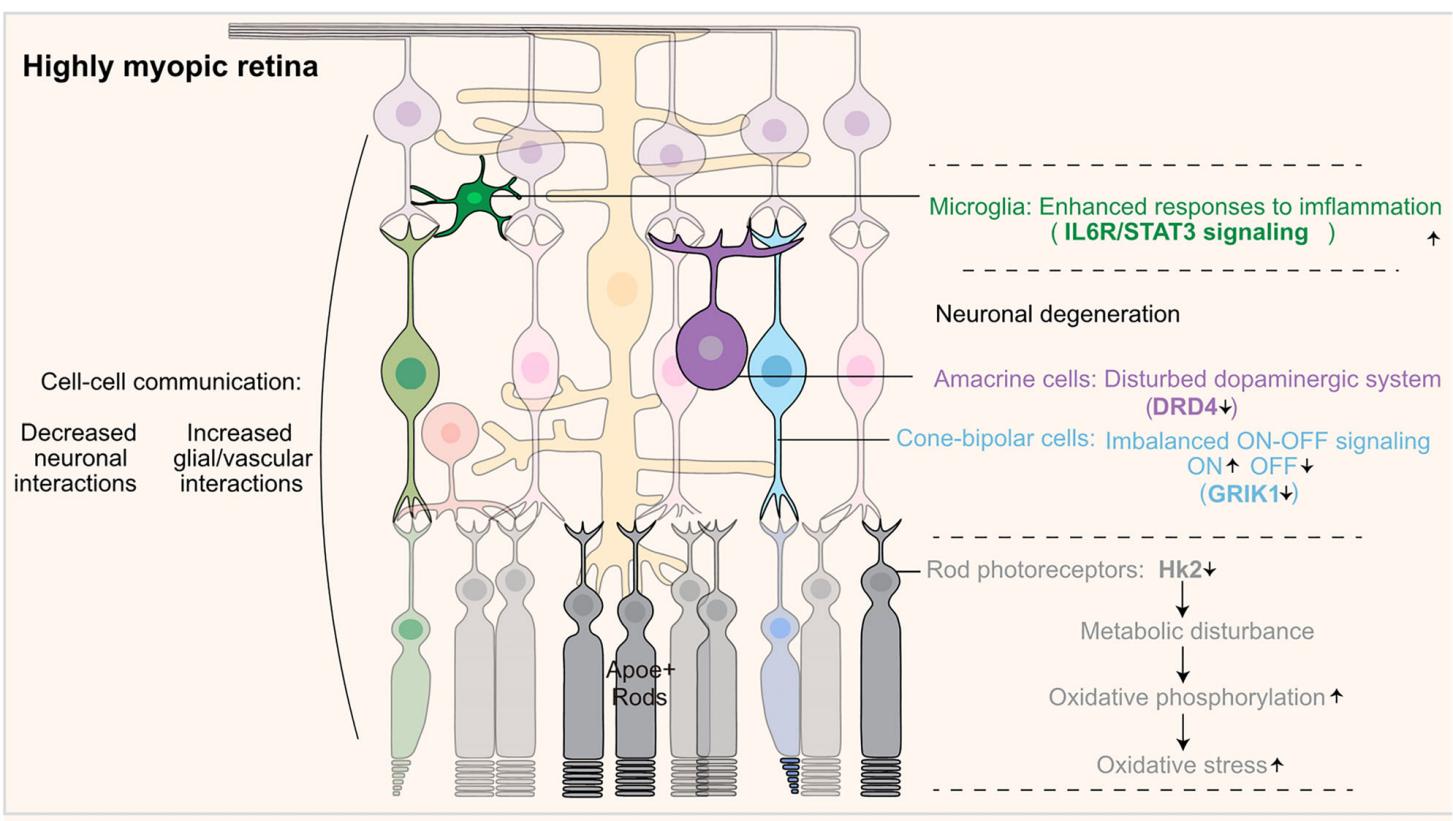
Schematic illustration of transcriptional landscape of retina in high myopia. In axially elongated highly myopic eyes, metabolic‐remodeling‐induced oxidative stress and a novel Apoe^+^ rod subpopulation was discovered, and aberrant light processing characterized by ON/OFF signaling imbalance driven by cone‐bipolar cells and dopamine receptors (*Drd4*) downregulation in amacrine cells were found. In addition, highly myopic microglia exhibited enhanced responses to cytokines. Furthermore, cell–cell communication network revealed attenuated neuronal interactions and increased glial/vascular interactions in highly myopic eyes. These results indicated a specific degeneration mode characterized by metabolic aberration, neuronal dysfunction, and increased inflammatory responses in highly myopic retinas, and revealed the cell‐type specific mechanisms underlying disease progression.

In conclusion, we have provided an outline of the transcriptional landscape of highly myopic retinas and revealed their generalized retinal neurodegeneration and enhanced responses to pathogenic factors (Figure 7). Our results imply that multiple retinal cell types are involved in the development of high myopia development. We have also identified a subpopulation of high‐myopia‐specific degenerative rods and a loss of balance between cone‐BC‐driven ON/OFF visual pathways, providing novel perspectives of the retinal mechanisms and some potential therapeutic targets in high myopia.

## 4 MATERIALS AND METHODS

### 4.1 Animals

Mice were obtained from SLAC Laboratory Animal Co. Ltd. and reared in specific pathogen‐free (SPF) barrier systems under a consistent 12‐h light/dark cycle (light on at 7:00 am and off at 7:00 pm). The humidity was kept at 40%−60% and the room temperature was kept at 21°C.

A −25.00 D lens was placed to the right eye of 4‐week‐old male C57BL/6 J mice to construct the defocus‐induced high myopia model. At the beginning of the study, mice with interocular baseline refraction of more than 1.00 D measured by a mouse infrared photorefractor (Steinbeis Transfer Center) were excluded. After 4 weeks of defocus induction, mice with the right eye showing at least 6.00 D more myopic than the left eye (self‐control) were considered an effective model of high myopia.

### 4.2 Retinal cells dissociation and single‐cell RNA sequencing

A total of three highly myopic retina samples and three control samples were prepared separately for sc‐RNA seq, and each sample was composed of five biologically duplicated retinas. The retinas were dissected in DMEM (Thermo Fisher Scientific) on ice, and then chopped up with scalpels and digested in Earle's balanced salt solution containing papain (20 units/mL) and DNAse I (2,000 units/mL) for 20 min at 37°C. Dissociation was stopped with ovomucoid. Cells were filtered through a 40 μm cell strainer (Bel‐Art), centrifuged (300 g, 5 min), and resuspended in Earle's balanced salt solution with 10% FBS. Trypan blue staining was used to evaluate cell concentration and viability. Cells from the six samples were then added to six respective channels on Chromium Single Cell Controller (10× Genomics). ScRNA‐seq libraries were constructed on 10× Genomics system and sequenced on NovaSeq 6000 (Illumina) after quality tests.

### 4.3 Data preprocessing, quality control, and sc‐RNA seq analysis

Raw sequencing data were mapped to the mm10 mouse reference genome using the 10× Genomics CellRanger software (version 5.0.0). The expression matrixes were processed using the Seurat package (version 4.1.0) in R software (version 4.1.2). Cells with RNA counts ≥1000, expressed genes ≥200, log_10_(genes per unique molecular identifier [UMI]) > 0.7, and mitochondrial RNA <25% were retained as high‐quality cells. Doublets were further detected using the DoubletFinder package (version 4.1.2) and removed from following analyses. After scaling and normalization, principal component analysis was performed using the top 2000 highly variable genes. Cell clustering was performed with the top 30 principal components at a resolution of 0.4 using the “FindClusters” function and visualized using Uniform Manifold Approximation and Projection (UMAP) or t‐Distributed Stochastic Neighbor Embedding (t‐SNE). Cell types were annotated using well‐established marker genes, and sub‐clustering would further be performed if one cluster was found to be mixed with multiple cell types.

### 4.4 Differentially expressed genes identification and functional enrichment analysis

Differentially expressed genes were generated with use of the “FindMarkers” function in Seurat package. Heat maps, volcano plots, and violin plots were generated with R software. Gene Ontology and Kyoto Encyclopedia of Genes and Genomes functional enrichment analyses were performed using the Metascape tool (http://metascape.org).^49^

### 4.5 Gene set variation analysis

The gene set variation analysis (GSVA) algorithm was used to evaluate the enrichment scores of interested pathways/gene sets among different cell types and groups. Heat map was used to visualize the GSVA results.

### 4.6 Pseudotime analysis

The Monocle2 package (version 2.8.0) was used to calculate the pseudotime trajectory of rod photoreceptors with unsupervised approaches.^50^ The start point of the pseudotime trajectory was determined by cell states. DDRTree was applied to reduce dimensions, and the “plot_cell_trajectory” function was used to visualize the cell trajectories.

### 4.7 Cell–cell communication analysis

To investigate the potential interactions between cell types, cell–cell communication analysis was performed using the CellPhoneDB Python package (version 1.0.0) based on expression of known interacting ligand‐receptor pairs.^51^ Two cell types were interacted if one cell type expressed the ligand and the other cell type expressed the receptor. “Expressed” was defined as at least 10% of cells in the specific cell‐type having nonzero read counts for the corresponding receptor or ligand genes.

### 4.8 Cell culture and treatment paradigms

The photoreceptor cell line, 661 W, and primary mouse RPE cells were both cultured in DMEM/F12 medium with 10% FBS and 1% penicillin and streptomycin. All cell cultures were maintained in a humidified environment of 95% air and 5% CO_2_ at 37°C. For *Hk2* knockdown, 661 W cells were transfected with siRNA (#siG190508023415; Guangzhou Ribo Company) using LipofectamineTM 3000 Reagent (Thermo Fisher Scientific) for 72 h. For ROS detection, cells were incubated in DCFH‐DA (#S0033; Beyotime) at 37°C for 30 min, washed by DPBS for three times, and then observed using a fluorescence microscope. For the co‐culture experiments, the 661 W cells were cultured in six‐well plates, and pre‐exposed to 0.005 mmol/L hydrogen peroxide (H_2_O_2_, #349887; Sigma) for 24 h before the Transwell inserts seeded with RPE cells were placed onto the plates.^52^

### 4.9 Metabolomic assays

To evaluate the glycolysis and OXPHOS levels, 661 W cells were plated onto Seahorse 96‐well plates. Cells were transfected with *Hk2* siRNAs or negative control siRNAs before being washed and incubated in the base medium (Agilent Technologies) at 37°C for 1 h. ECAR and OCR were measured by glycolysis stress tests and mitochondria stress tests on seahorse Bioscience X96 extracellular flux analyzer (Agilent Technologies) according to the manufacturer's instructions, serving as quantitative indicators for cellular glycolysis and OXPHOS levels, respectively.^34^

### 4.10 Western blotting

Protein was extracted from retinas or cultured cells, separated on 10% SDS‐PAGE gel, and transferred to nitrocellulose membranes. After blockage, the membranes were incubated with primary antibodies at 4°C overnight, followed by incubating with HRP‐conjugated secondary antibodies at room temperature for 1 h. After visualization with ECL Plus Western Blotting Substrate (Thermo Fisher Scientific) and ECL detection system (ChampChemi, SAGECREATION), the density of target protein bands was evaluated using the ImageJ software and normalized to the loading control (GAPDH) before statistical analysis. The following primary antibodies were used for western blotting: GRIK1 (GLUR5, 1:100, #sc‐393420; Santa Cruz), HK2 (1:2000, 22029‐1‐AP; ProteinTech), MMP2 (1:500, #10373‐2‐AP; ProteinTech), STAT3 (1:1000, #9139; Cell Signaling Technology), p‐STAT3 (1:1000, #9145; Cell Signaling Technology), and GAPDH (1:3000, #5174; Cell Signaling Technology).

### 4.11 Immunofluorescence

Frozen sections of mice eyes were blocked, incubated with primary antibodies at 4°C overnight, and then incubated with secondary antibodies and DAPI for 2 h. Slides were observed using a confocal microscope (Leica Microsystems). The primary antibodies used for immunofluorescence were as follows: PRKCA (1:200, #P4334; Sigma Aldrich), APOE (1:50, #66830‐1‐lg; ProteinTech), p‐STAT3 (1:200, #9145; Cell Signaling Technology), and IBA1 (1:200, #ab283319; Abcam).

### 4.12 Statistics

The experimental data between highly myopic and self‐control eyes were compared using paired *t*‐test. Other experimental data between two groups were compared using independent sample *t*‐test. *p* Value < 0.05 was considered statistically significant.

## AUTHOR CONTRIBUTIONS

Yunqian Yao and Xiangjia Zhu conceived the idea and wrote the initial and revised versions of the manuscript. Xingtao Zhou supervised the study and was involved in critical revision of manuscript. Yi Lu provided material support. Qingfeng Wu provided technical support. Yunqian Yao and Zhenhua Chen performed bioinformatic analyses and experiments. All authors reviewed the results and approved the final version of the manuscript.

## CONFLICT OF INTEREST STATEMENT

The authors declare no conflicts of interest.

## ETHICS STATEMENT

All the animal procedures conformed to the ARVO Statement and were approved by the Ethics Committee for Animal Studies of Eye & ENT Hospital of Fudan University (approval number, 2020068).

## Supporting information

Supporting Information

## Data Availability

The data that support the findings of this study are available from the corresponding author upon reasonable request.
