# Supplementary material for: Single‐cell RNA sequencing of retina revealed novel transcriptional landscape in high myopia and underlying cell‐type‐specific mechanisms
Source: MedComm (2020). 2023 Sep 20;4(5):e372. doi: 10.1002/mco2.372 (PMC10511833; doi:10.1002/mco2.372)
Supplement: Supplementary file 1 — Supporting Information [file MCO2-4-e372-s001.docx]

**Supplementary Materials for**

**Single-cell RNA sequencing of retina revealed novel transcriptional landscape in high myopia and underlying cell-type-specific mechanisms**

Yunqian Yao, ^1,2,3^ Zhenhua Chen, ^4,5^ Qingfeng Wu, ^4,5,6,7,8^ Yi Lu, ^1,2,9,10*^ Xingtao Zhou, ^1,2,3*^ Xiangjia Zhu ^1,2,9,10*^

**Institutions:**

1. Eye Institute and Department of Ophthalmology, Eye & ENT Hospital, Fudan University, Shanghai, China.

2. National Health Center Key Laboratory of Myopia (Fudan University), Key Laboratory of Myopia, Chinese Academy of Medical Sciences, Shanghai, China.

3. Shanghai Research Center of Ophthalmology and Optometry, Shanghai, China.

4. State Key Laboratory of Molecular Development Biology, Institute of Genetics and Developmental Biology, Chinese Academy of Sciences, Beijing 100101, China

5. University of Chinese Academy of Sciences, Beijing 100101, China

6. Center for Excellence in Brain Science and Intelligence Technology, Chinese Academy of Sciences, Beijing 100101, China

7. Chinese Institute for Brain Research, Beijing 102206, China

8. Beijing Children’s Hospital, Capital Medical University, Beijing 100045, China

9. Shanghai Key Laboratory of Visual Impairment and Restoration, Shanghai, China.

10. State Key Laboratory of Medical Neurobiology, Fudan University.

**Correspondence:**

Xiangjia Zhu, Xingtao Zhou and Yi Lu, Eye & ENT Hospital of Fudan University, 83 Fenyang Road, Shanghai 200031, China.

E-mail: [zhuxiangjia1982@126.com](mailto:zhuxiangjia1982@126.com); doctzhouxingtao@163.com; luyieent@163.com


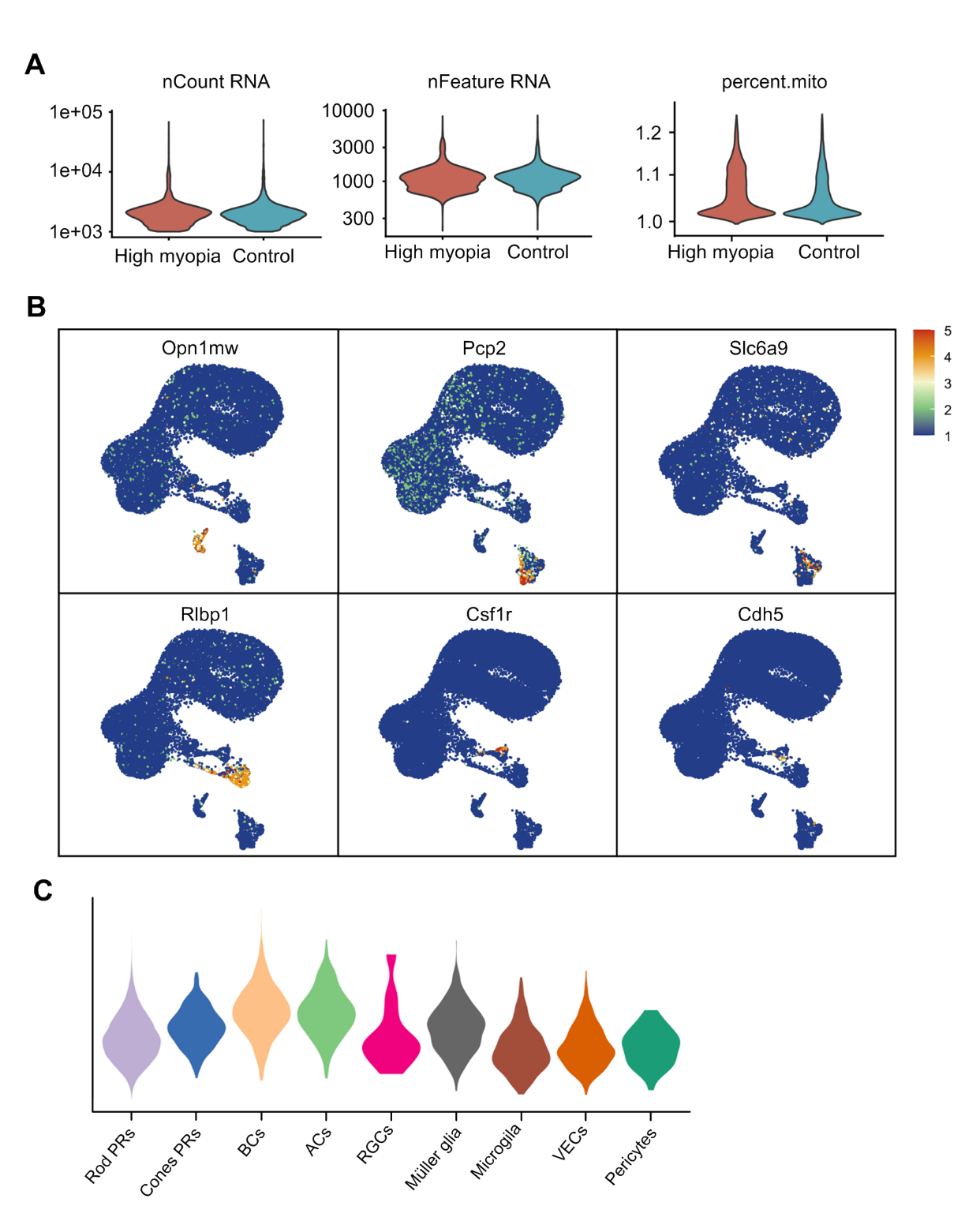


**Figure S1. (A)** Violin plots showing the number of genes per cell (nFeature RNA), UMIs per cell (nCount RNA) and the proportion of mitochondrial genes (percent.mt) in high myopia and control groups after quality control. **(B)** Feature plots showing the expression patterns of marker genes of retinal cell-types (*Opn1mw*, cone photoreceptors; *Opn1mw*, cone photoreceptors; ). **(C)** Violin plot showing the scores of each retinal cell type for their expression of myopia candidate genes.


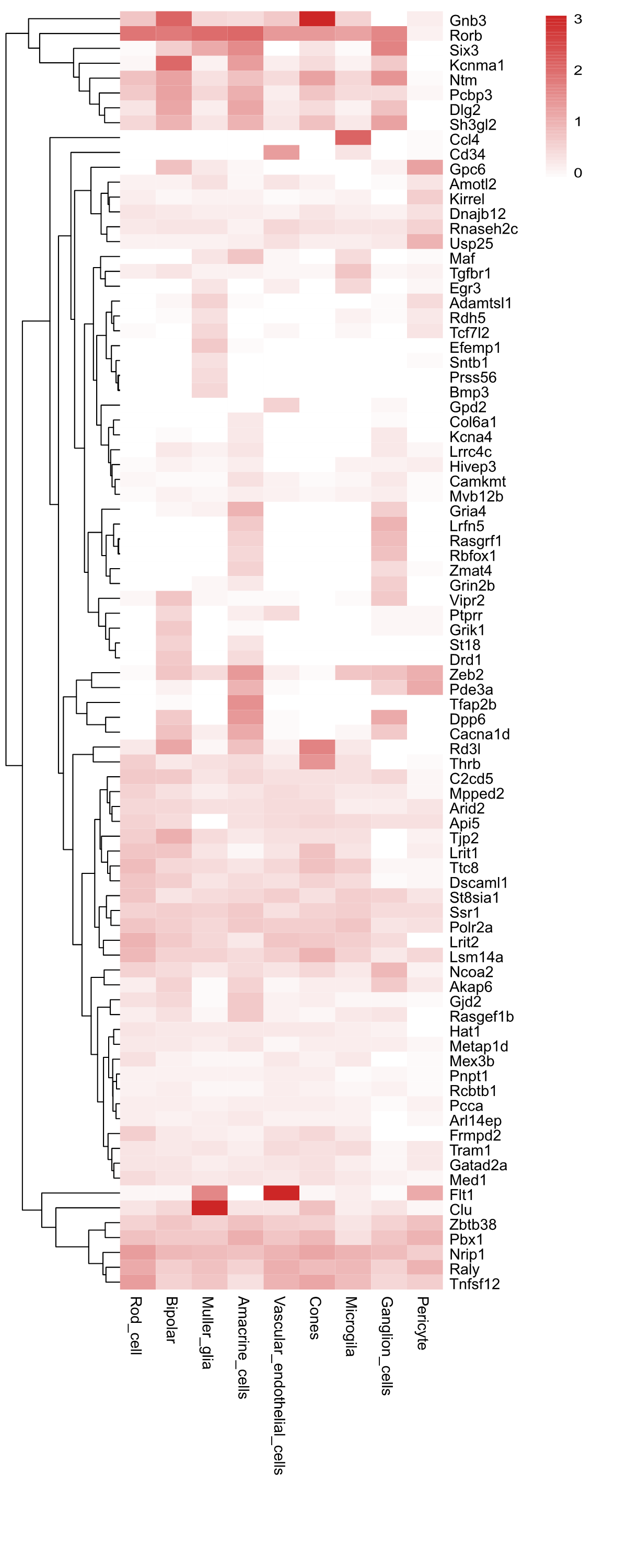


**Figure S2.** Expression profiles of myopia candidate genes in each retinal cell type.


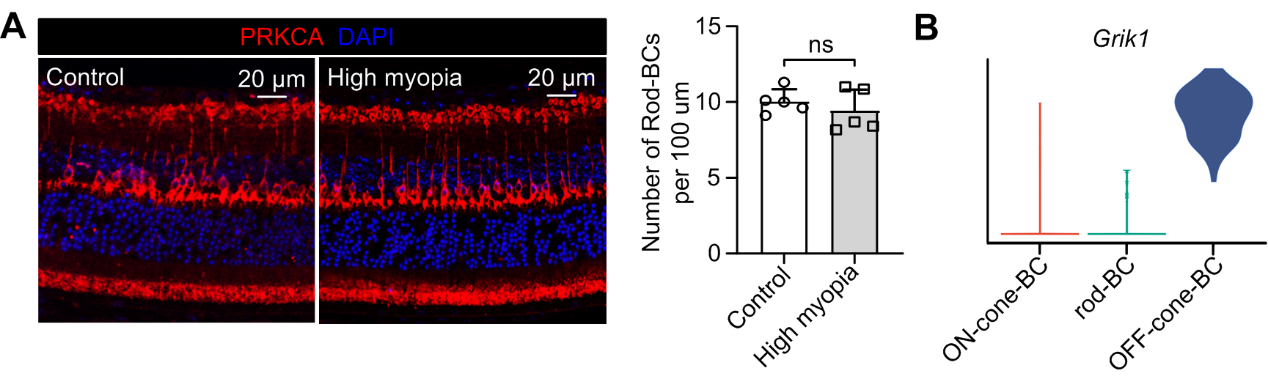


**Figure S3. (A)** Immunofluorescence staining for rod BCs showed no significant difference in density/proportion between highly myopic and control eyes. **(B)** Violin plot showing that Grik1 was specifically expression in OFF-cone-BCs.
